# Regulation of tumor suppressor EAF2 polyubiquitination by ELL1 and SIAH2 in prostate cancer cells

**DOI:** 10.18632/oncotarget.8588

**Published:** 2016-04-05

**Authors:** Xinpei Yu, Junkui Ai, Liquan Cai, Yifeng Jing, Dan Wang, Jun Dong, Laura E. Pascal, Jian Zhang, Rongcheng Luo, Zhou Wang

**Affiliations:** ^1^ Department of Urology, University of Pittsburgh School of Medicine, Pittsburgh, USA; ^2^ Department of Pathology, University of Pittsburgh School of Medicine, Pittsburgh, USA; ^3^ Department of Pharmacology and Chemical Biology, University of Pittsburgh School of Medicine, Pittsburgh, USA; ^4^ University of Pittsburgh Cancer Institute, University of Pittsburgh School of Medicine, Pittsburgh, USA; ^5^ Department of Geriatrics, Guangzhou General Hospital of Guangzhou Military Command, Guangzhou, China; ^6^ Department of Urology, Shanghai General Hospital, Shanghai Jiao Tong University School of Medicine, Shanghai, China; ^7^ Center for Translational Medicine, Guangxi Medical University, Nanning, Guangxi, China; ^8^ Cancer Center, Traditional Chinese Medicine-Integrated Hospital, Southern Medical University, Guangzhou, China; ^9^ Guangdong Provincial Key Laboratory of Geriatric Infection and Organ Function Support and Guangzhou Key Laboratory of Geriatric Infection and Organ Function Support, Guangzhou, China

**Keywords:** prostate cancer, EAF2, ELL, SIAH2, ubiquitination

## Abstract

RNA Polymerase II Elongation Factor (ELL)-associated factor 2 (EAF2) is a tumor suppressor frequently down-regulated in human prostate cancer. We previously reported that its binding partner ELL1 can enhance EAF2 protein stability and activity. Here we show that EAF2 can be polyubiquitinated and its degradation blocked by proteasome inhibitor. Co-immunoprecipitation detected EAF2 binding to SIAH2, an E3 ligase, and SIAH2 overexpression enhanced polyubiquitination of EAF2. Co-transfection of EAF2 binding partner ELL1 blocked EAF2 ubiquitination, providing a mechanism for EAF2 stabilization. Finally, EAF2K81R mutant, which exhibits reduced polyubiquitination and increased stability, was more potent than wild-type EAF2 in apoptosis induction. These findings suggest that SIAH2 is an E3 ligase for EAF2 polyubiquitination and ELL1 can enhance EAF2 level and function by blocking its polyubiquitination.

## INTRODUCTION

Prostate cancer is a major cause of cancer death in aging males, particularly in Western countries [1]. Elucidating the mechanisms involved in prostate carcinogenesis is clinically relevant and may lead to new approaches for the prevention and/or treatment of the disease. In the past two decades, inactivation of multiple tumor suppressors was reported to promote prostate carcinogenesis (Reviewed in [2]). RNA Polymerase II Elongation Factor (ELL)-associated factor 2 (EAF2) is one of the tumor suppressors involved in prostate carcinogenesis [3–9].

EAF2 is encoded by an androgen upregulated gene 19 (U19) [9], which was initially identified from the rat ventral prostate model [10]. EAF2 and its homolog, EAF1, are positive regulators of RNA polymerase II elongation factor ELL1 [11]. Immunostaining revealed EAF2 downregulation in ~80% high Gleason grade human prostate cancer specimens and in all tested prostate cancer cell lines [9]. Overexpression of EAF2 induced apoptosis in cultured prostate cancer cells as well as in prostate cancer xenograft tumors [9], and EAF2 knockdown in LNCaP cells enhanced the expression of androgen receptor (AR)-target genes, cell proliferation, and migration [12]. Knockout of EAF2 gene in mice led to the development of high grade prostatic intraepithelial neoplasia, the putative precursor of prostate cancer [8]. When EAF2 knockout was combined with PTEN heterozygous deletion, the double knockout mice developed prostate cancer [3]. These observations indicate that EAF2 is a tumor suppressor in the prostate.

The turnover of many important tumor suppressors is regulated, which represents a major mechanism to control their activities [13, 14]. The binding of ELL1 to EAF2 enhances EAF2 protein level [7], suggesting that EAF2 protein turnover can also be regulated. However, the mechanisms regulating EAF2 protein turnover have not been elucidated. ELL2, a homolog of ELL1, was reported to undergo polyubiquitination and proteasomal degradation via the RING domain protein SIAH1 as the E3 ubiquitin ligase [15, 16]. Since EAF2 is a binding partner of ELL2, EAF2 protein turnover may also be regulated by a similar mechanism.

In the present paper, we investigated the regulation of EAF2 protein turnover by polyubiquitination and the regulation of EAF2 polyubiquitination by its binding partners ELL1 and ELL2. Furthermore, we tested the potential role of SIAH1 and SIAH2 in EAF2 polyubiquitination. Our results provided new insights into the mechanisms regulating EAF2 protein turnover, which may eventually lead to novel approaches to stabilize EAF2 and subsequently enhance its tumor suppressive activity in prostate cancer.

## RESULTS

### Proteasome inhibition enhanced EAF2 protein stability in prostate cancer cells

**Figure 1:**
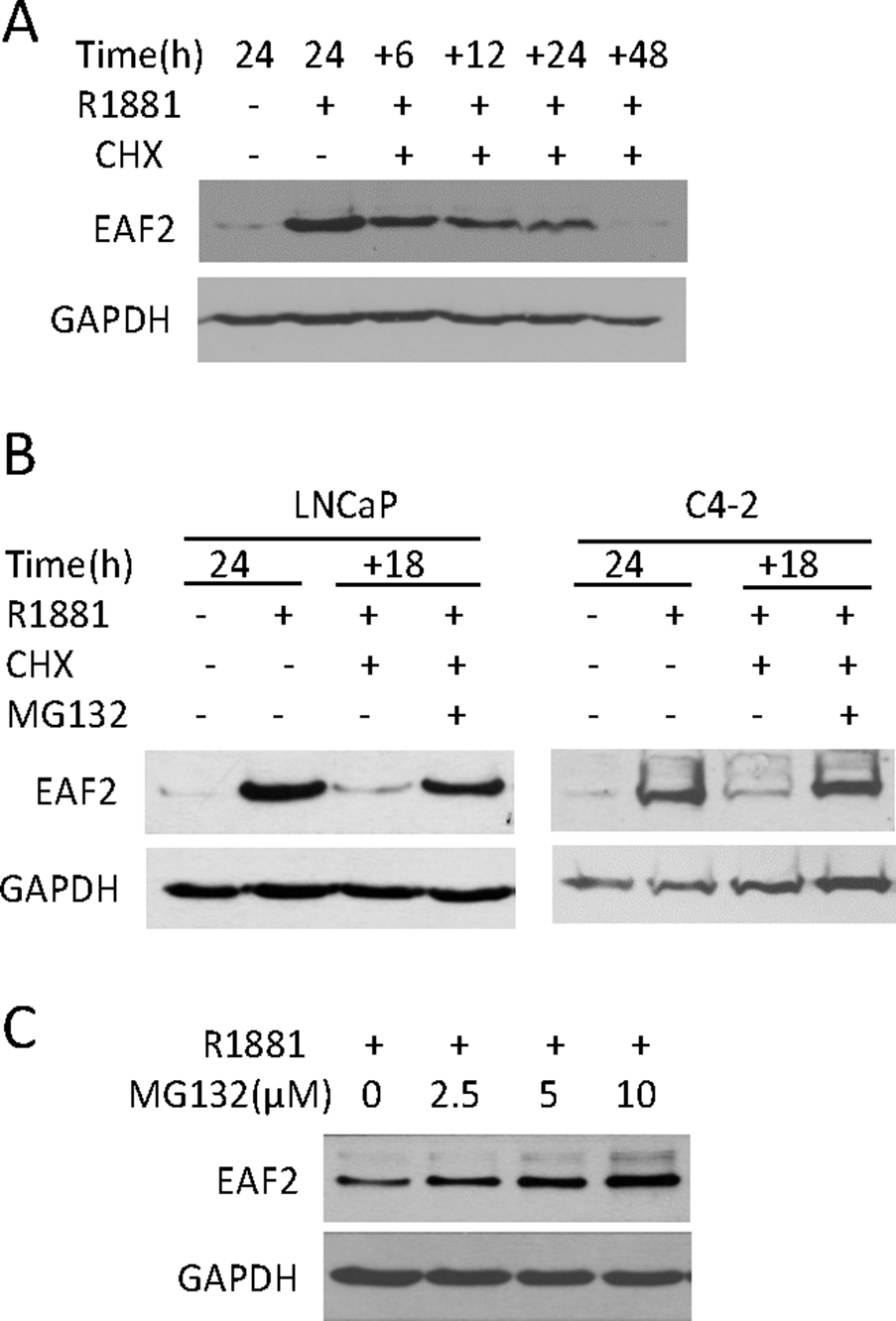
The effect of cyclohexamide (CHX) and/or MG132 on endogenous EAF2 protein level in prostate cancer cells (**A**) Western blot analysis of EAF2 in C4-2 cells treated with 1 nM R1881 for 24 h, and then treated with CHX at 50 μg/ml for an additional 6, 12, 24, or 48 h. (**B**) Western blot analysis of EAF2 in LNCaP and C4-2 cells cultured for an additional 18 h in the presence or absence of 50 μg/ml CHX and/or 5 μM MG132. (**C**) Western blot analysis of EAF2 in C4-2 cells treated with indicated concentrations of MG132 for an additional 18 h. All experiments were conducted in the presence of 1 nM R1881 24 h prior to treatment to stimulate EAF2 expression. GAPDH was probed as a loading control. Data shown are representative of three independent experiments.

EAF2 is an unstable protein with a short half-life [7, 17]. To evaluate EAF2 protein stability, AR-positive C4-2 prostate cancer cells [18] were cultured in the presence of synthetic androgen R1881 for 24 hours to induce EAF2 expression and then treated with protein synthesis inhibitor, CHX for 6 to 48 hours (Figure 1A). Western Blot analysis showed that about half of the EAF2 protein remained 6 hours after CHX treatment and EAF2 level continued to decrease and virtually disappeared 48 hours after CHX treatment, suggesting that EAF2 was not stable, with a half-life of about 6 hours in C4-2 cells.

Since proteasome can degrade proteins [13, 19], we tested whether proteasome inhibition could block EAF2 degradation in the presence of CHX. The presence of proteasome inhibitor, MG132, significantly blocked EAF2 protein decay in the presence of CHX in both LNCaP and C4-2 prostate cancer cell lines (Figure 1B). In addition, MG132 increased the protein level of EAF2 in C4-2 in a dose-dependent manner in the presence of 1 nM R1881 (Figure 1C). These observations suggest that proteasome is required for EAF2 degradation.

### EAF2 protein undergoes polyubiquitination

**Figure 2:**
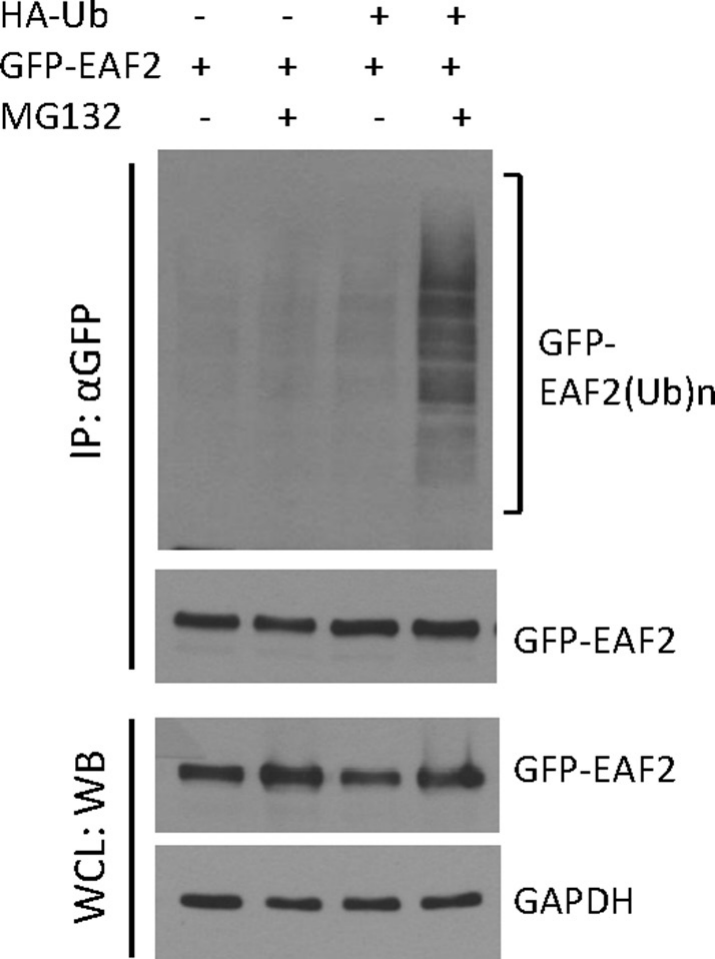
Polyubiquitination of GFP-EAF2 protein HEK 293 cells were transiently transfected with GFP-EAF2 expression vector in the presence or absence of HA-ubiquitin vector for 30 h, and then treated with 10 μM MG132 or vehicle control for additional 18 h. GFP-EAF2 was isolated from the denatured cell lysates using anti-GFP antibody, followed by immunoblotting using both anti-GFP and anti-ubiquitin antibodies. The whole cell lysates (WCL) was probed with anti-GFP antibody to determine the expression of GFP-EAF2. GAPDH was probed as loading control. Data shown are representative of three independent experiments.

**Figure 3:**
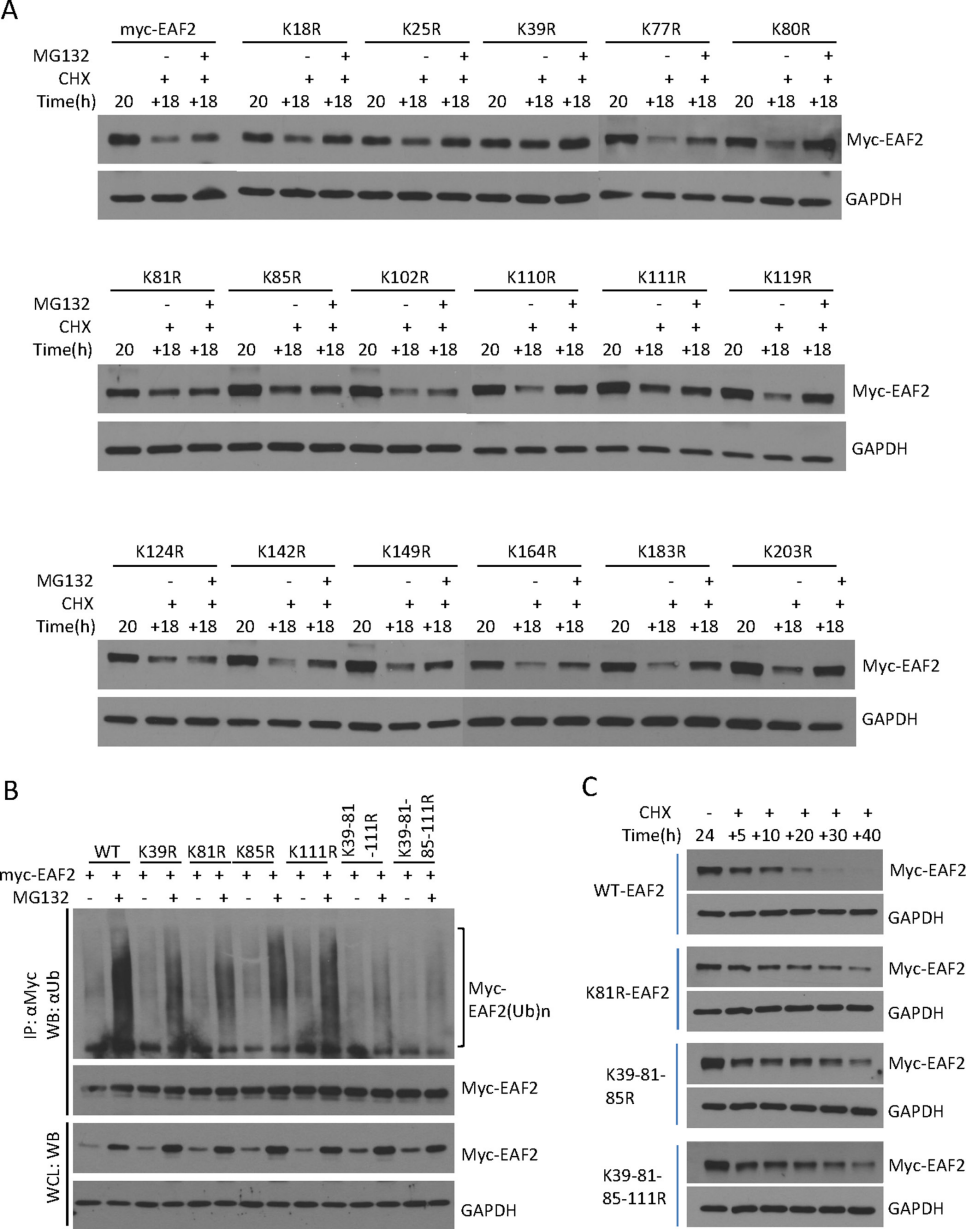
Identification of ubiquitination sites of EAF2 protein (**A**) C4-2 cells were transfected for 24 h with wild-type myc-EAF2 or myc-tagged EAF2 mutants with following single lysine substitution with arginine: K18R, K25R, K39R, K77R, K80R, K81R, K85R, K102R, K110R, K111R, K119R, K124R, K142R, K149R, K164R, K183R, and K203R. The cells were treated with CHX (50 μg/ml) in the presence or absence of MG132 (5 μM) for an additional 18 h. Cell lysates were analyzed by immunoblotting using anti-myc and anti-GAPDH antibodies. (**B**) HEK 293 cells were co-transfected with HA-ubiquitin and wild-type myc-EAF2, K39R, K81R, K85R, K111R, K39-81-111R or K39-81-85-111R mutant EAF2. After 30 h, cells were treated with or without MG132 (10 μM) for an additional 18 h. Myc-EAF2 was isolated from denatured cell lysates by immunoprecipitation using anti-myc antibody, followed by immunoblotting of the precipitates. Myc-EAF2 and HA-ubiquitin were detected using anti-myc and anti-ubiquitin antibodies, respectively. (**C**) C4-2 cells were transfected with wild-type myc-EAF2, K81R mutant EAF2, K39-111-85R mutant EAF2 or K39-111-81-85R mutant EAF2 vectors individually for 20 h. Cells were then treated with CHX (50 μg/ml) for additional 5, 10, 20, 30 and 40 h. Whole cell lysates were analyzed by immunoblotting for myc-EAF2 using anti-myc antibody. GAPDH was immunoblotted as a loading control. Data shown are representative of three independent experiments.

Proteins targeted for degradation by the proteasome are polyubiquitinated [13, 20]. We used HEK293 cells to study EAF2 polyubiquitination because the transfection efficiency in HEK293 cells is much higher than in prostate cancer cells. Polyubiquitinated GFP-EAF2 was readily detected in the presence of HA-ubiquitin and MG132 (Figure 2). Similar findings were observed using myc-EAF2 expression vector (data not shown). These observations suggest that polyubiquitination is involved in proteasome-dependent EAF2 degradation. Since ubiquitination typically occurs at lysine residues, we generated 17 single substitution mutants by replacing lysine with arginine individually in the EAF2 protein in order to determine the potential sites of ubiquitination. Subsequently, we tested EAF2 stability in the presence of CHX or both CHX and MG132 (Figure 3A). The substitution mutants, K39R, K81R, K85R, and K111R exhibited a higher level than wild-type EAF2 and other substitution mutants in the presence of CHX. Also, among all the mutants tested, K81R appeared to be the least sensitive to MG132 when CHX was present. This finding suggests that K39, K81, K85 and K111 are likely the major sites for polyubiquitination of EAF2, with K81 being the most important.

As expected, EAF2 mutants with individual substitution at the K39, K81, K85 or K111 exhibited reduced polyubiquitination (Figure 3B), suggesting that each of these sites could be polyubiquitinated. Among the single substitution EAF2 mutants, K81R appeared to have the most significant reduction in polyubiquitination. Triple substitution mutant K39-81-111R and quadruple substitution mutant K39-81-85-111R had a more dramatic inhibition of EAF2 polyubiquitination (Figure 3B), further indicating that K39, K81, K85, and K111 are major sites for EAF2 polyubiquitination. In the presence of CHX, EAF2 mutants K81R, K31-85-111R, and K31-81-85-111R were much more stable than wild-type EAF2. Wild-type EAF2 was barely detectable 30 hours after CHX treatment, whereas these mutant EAF2 proteins were still present, even 40 hours after CHX treatment (Figure 3C). These findings argue that K39, K81, K85 and K111 are sites for EAF2 ubiquitination, with K81 being the most important site.

**Figure 4:**
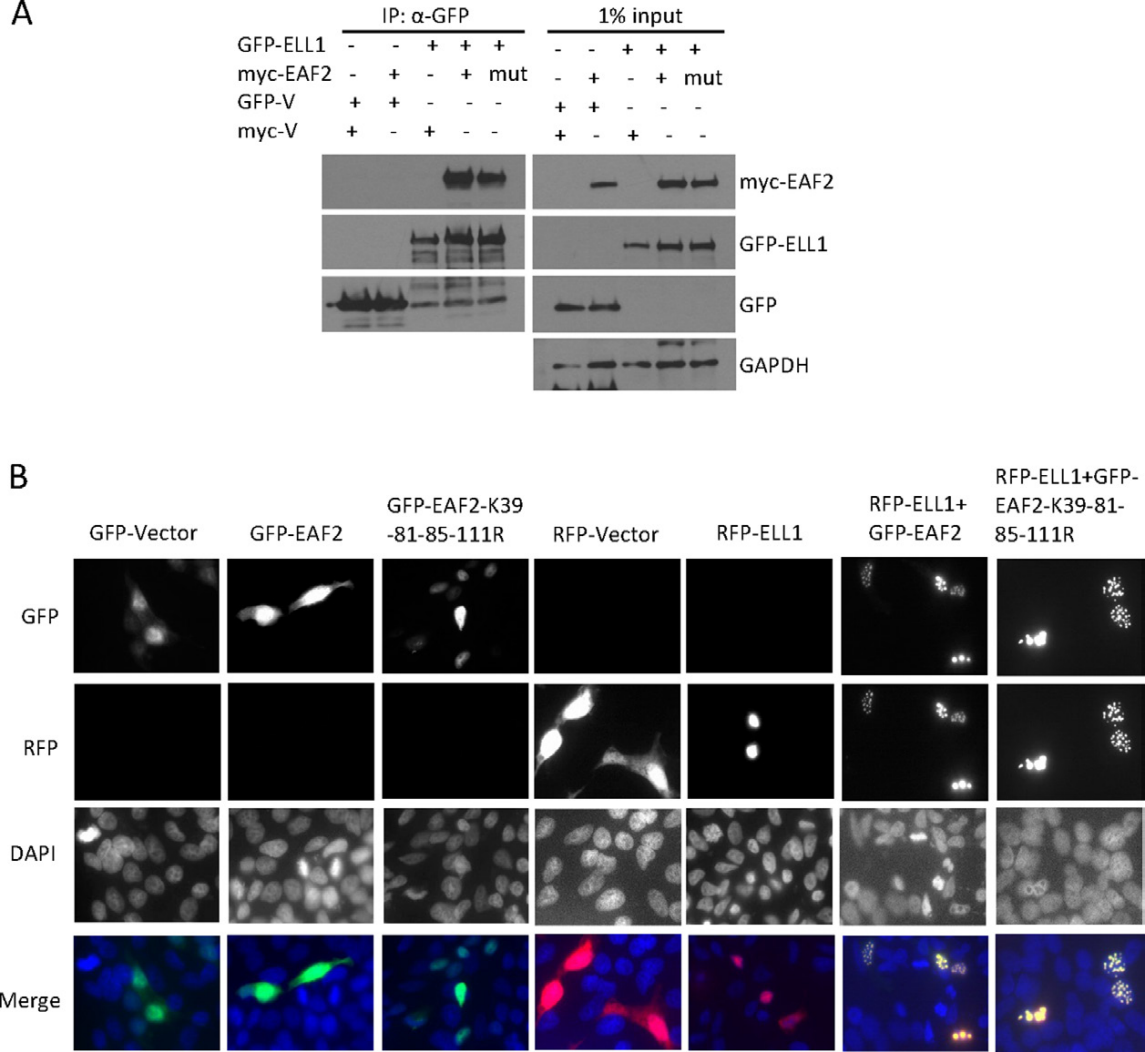
Mutant EAF2^K39-81-85-111R^ binding and co-localization with ELL1 (**A**) HEK 293 cells were transfected with myc-EAF2, myc-EAF2^K39-81-85-111R^, or empty myc expression vector together with GFP-ELL1 or empty GFP expression vector for 36 h. The cell lysates were prepared for co-immunoprecipitation using anti-GFP antibody. The precipitates and whole cell lysates (1% input) were analyzed by immunoblotting using anti-myc and anti-GFP antibodies. GAPDH in the whole cell lysates was probed as loading control. (**B**) C4-2 cells were transfected with GFP, GFP-EAF2, GFP-EAF2^K39-81-85-111R^, RFP, and RFP-ELL1 expression vector alone or in the indicated combinations for 48 h. Subcellular localization was imaged with confocal microscopy. Image enlargement: 100×. Data shown are representative of three independent experiments.

The replacement of lysine to arginine in mutant EAF2 may have some effect on EAF2 3D structure, although both lysine and arginine are structurally similar. The quadruple substitution EAF2 mutant K39-81-85-111R retained the ability to bind ELL1, although co-immunoprecipitation of K39-81-85-111R with ELL1 appeared slightly less effective than that of wild-type EAF2 (Figure 4A). In the presence of ELL1, EAF2 localized into nuclear speckles [7, 17]. GFP-tagged EAF2 K39-81-85-111R formed nuclear speckles with RFP-ELL1, in a similar fashion to the GFP-tagged wild-type EAF2 (Figure 4B). These observations indicated that the lysine to arginine substitution caused some, but not significant, alteration in the structure of EAF2.

### SIAH2 binds to and enhances polyubiquitination of EAF2

**Figure 5:**
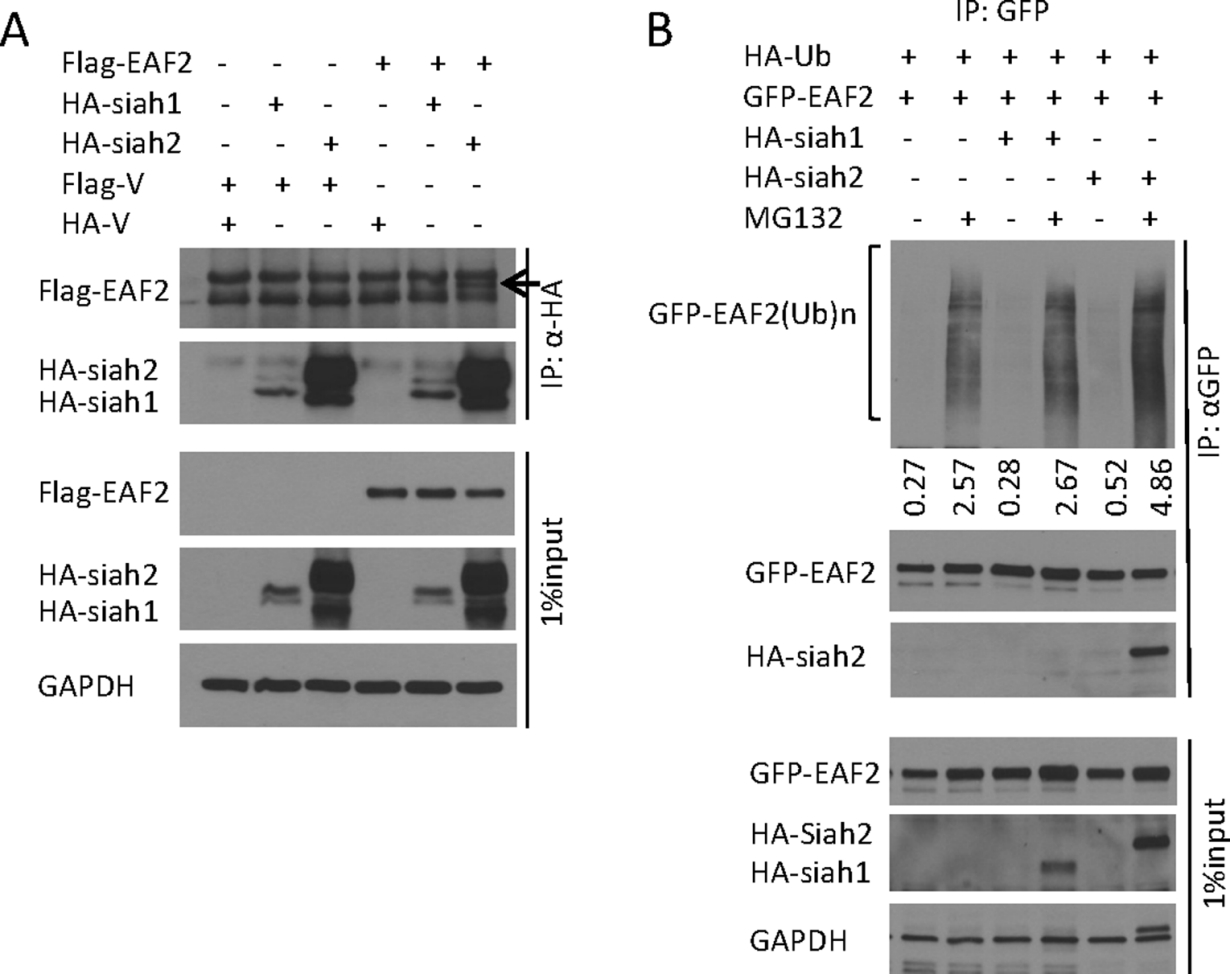
E3 ligase SIAH2 interaction with EAF2 protein (**A**) HEK 293 cells were transfected to express flag-EAF2, together with HA-tagged SIAH1 or SIAH2 for 30 h. Then cells were treated with MG132 (10 μM) for an additional 18 h. EAF2 was isolated from denatured cell lysates by anti-HA immunoprecipitation, followed by immunoblotting of the precipitates. The precipitated Flag-EAF2 (indicated by an arrow) and HA-SIAH1/2 were detected with anti-flag and anti-HA antibodies, respectively. Whole cell lysates were also immunoblotted to detect the expression of transfected flag-EAF2 and HA-SIAH1/2. (**B**) HEK 293 cells were co-transfected to express GFP-EAF2, HA-tagged ubiquitin, and HA-tagged SIAH1 or SIAH2 for 30 h. Then cells were treated with MG132 (10 μM) for an additional 18 h. GFP-EAF2 was isolated from denatured cell lysates by anti-GFP immunoprecipitation, followed by immunoblotting of the precipitates. GFP-EAF2 and HA-SIAH1/2 were detected with anti-GFP, anti-SIAH1 or SIAH2 antibodies, respectively. The numbers below the Western image indicate the relative intensity of polyubiquitinated GFP-EAF2, which were quantified using ImageJ (NIH). GAPDH was immunoblotted as a loading control. Data shown are representative of three independent experiments.

SIAH1 was reported as an E3 ligase of EAF2 binding partner ELL2 [15]. Proteins binding to ELL1 or ELL2 may also bind to EAF2. For example, p53, which was reported to bind to ELL1 [21], also binds EAF2 [5]. Since SIAH1 binds to ELL2 [15], we tested whether SIAH1 and its homolog SIAH2 bind to EAF2. Flag-EAF2 was co-immunoprecipitated by anti-HA antibody precipitation of HA-SIAH2 but not HA-SIAH1 (Figure 5A). Reciprocally, anti-GFP antibody precipitation of GFP-EAF2 also precipitated HA-SIAH2 but not HA-SIAH1 (Figure 5B). For unknown reasons, HA-SIAH1 expression level was much lower than HA-SIAH2 in our experiments (Figure 5A and 5B). The lower level of SIAH1 expression could make it difficult to detect potential binding between EAF2 and SIAH1. Thus, it is still possible for SIAH1 to act as an E3 ligase for EAF2.

We next tested whether SIAH1 and/or SIAH2 could enhance polyubiquitination of EAF2 protein. Co-transfection of HA-SIAH1 had no effect on EAF2 ubiquitination whereas co-transfection with HA-SIAH2 induced an 82% increase in ubiquitination of EAF2 (Figure 5B). This finding suggests that SIAH2 could function as an E3 ligase for EAF2. The inability of the transfected SIAH1 to enhance EAF2 ubiquitination may be due to its low level expression.

### ELL1, but not ELL2, blocks EAF2 ubiquitination

**Figure 6:**
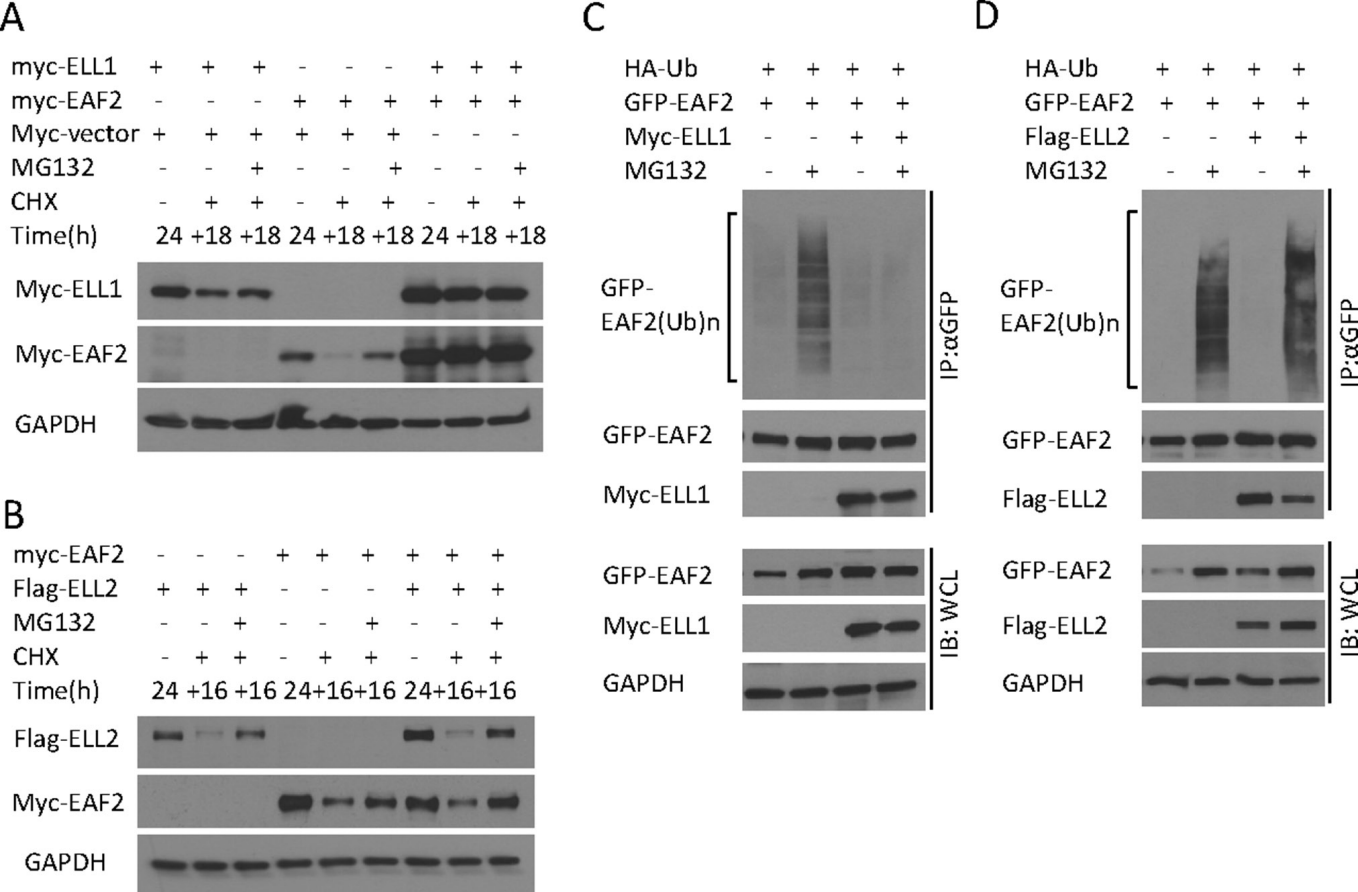
The effect of ELL1 and ELL2 on EAF2 stability and ubiquitination (**A**) Myc-EAF2, myc-ELL1 or empty myc vector were transfected into C4-2 cells alone or in combination as indicated for 24 h. Then cells were treated with CHX (50 μg/ml) with or without MG132 (5 μM) for additional 18 h. Cell lysates were analyzed by immunoblotting for EAF2 (anti-myc) and ELL1 (anti-myc). (**B**) Myc-EAF2, flag-ELL2 or empty myc vector were transfected into C4-2 cells alone or in combination for 24 h. Then cells were treated with CHX (50 μg/ml) with or without MG132 (5 μM) for additional 16 h. Cell lysates were analyzed by immunoblotting for EAF2 (anti-myc) and ELL2 (anti-Flag). (**C** and **D**) GFP-EAF2 and HA-ubiquitin were co-transfected into HEK 293 cells with or without the transfection of myc-ELL1 (C) or flag-ELL2 (D). After 30 h, cells were treated with or without MG132 (10 μM) for additional 18 h. EAF2 was isolated from denatured cell lysates by anti-GFP immunoprecipitation, followed by immunoblotting of the precipitates. HA-ubiquitin, GFP-EAF2, flag-ELL2 and myc-ELL1 were detected by anti-ubiquitin, anti-GFP, anti-flag and anti-myc antibodies, respectively. GAPDH was immunoblotted as a loading control. Data shown are representative of three independent experiments.

Co-transfection of ELL1 in C4-2 cells enhanced the level of EAF2 and inhibited EAF2 degradation in the presence of CHX (Figure 6A). Since ELL2 is a major EAF2 binding partner in the prostate, we also tested the effect of ELL2 co-transfection on EAF2 stability. Unlike myc-ELL1, Flag-ELL2 overexpression was unable to prevent the degradation of myc-EAF2 in the presence of CHX (Figure 6B). Since EAF2 stability was reduced by polyubiquitination and proteasome, we tested whether ELL1, but not ELL2, could suppress EAF2 ubiquitination. Co-transfection of myc-ELL1 inhibited ubiquitination of GFP-EAF2 in HEK 293 cells (Figure 6C). In contrast, co-transfection of Flag-ELL2 did not inhibit the ubiquitination of GFP-EAF2 (Figure 6D). These findings suggest that ELL1, but not ELL2, can inhibit polyubiquitination and subsequently proteasome-dependent degradation of EAF2.

### EAF2^K81R^ mutant is more potent than wild-type EAF2 in apoptosis induction

**Figure 7:**
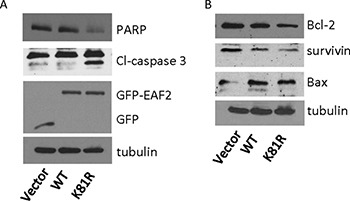
Effect of K81R substitution on EAF2 induction of apoptosis HEK 293 cells were transiently transfected with GFP, GFP-EAF2 or GFP-EAF2^K81R^ for 72 h. Cells lysates were analyzed by immunoblotting using anti-PARP, anti-cleaved (Cl)-caspase 3 and anti-GFP antibodies for panel (**A**) and using anti-Bcl-2, anti-survivin and anti-Bax antibodies for panel (**B**) Tubulin was probed as loading control. Data shown are representative of three independent experiments.

To evaluate whether ubiquitination of EAF2 could inhibit its function, we tested whether K81R substitution could enhance the pro-apoptotic activity of EAF2. Compared to single substitution mutations, triple or quadruple substitutions are more likely to alter the 3D structure of EAF2, in addition to inhibiting polyubiquitination. Thus, we did not test triple or quadruple substitution mutants in this study. EAF2^K81R^ was used here since K81R was the most effective single substitution mutation in inhibiting polyubiquitination (Figure 3B). GFP-EAF2 or GFP-EAF2^K81R^ was transfected into HEK 293 cells due to their high transfection efficiency. The lysates of transfected cells were analyzed by Western Blot for markers associated with apoptosis including cleaved-caspase 3, PARP, BCL2, survivin, and BAX [22–25] (Figure 7). As compared to GFP-EAF2, GFP-EAF2^K81R^ transfection increased cleaved-caspase 3, reduced full-length PARP and Bcl-2, and also slightly reduced the levels of survivin and Bax. This result suggests that EAF2^K81R^ is more potent than wild-type EAF2 in inducing apoptosis, potentially due to its increased stability.

## DISCUSSION

The present study provides evidence for polyubiquitination of tumor suppressor EAF2 and identified SIAH2 as an E3 ligase for EAF2. We showed inhibition of EAF2 polyubiquitination by the binding of ELL1, but not ELL2. Furthermore, EAF2^K81R^, a mutant EAF2 with reduced polyubiquitination, was more potent than wild-type EAF2 in apoptosis induction. These observations suggested that EAF2 protein turnover is enhanced by polyubiquitination, which can be catalyzed by SIAH2 and inhibited by ELL1, and that inhibition of EAF2 polyubiquitination could enhance EAF2 activity.

The expression of EAF2 is induced by androgens in the prostate [9, 10]. Interestingly, the expression of its binding partner ELL2 is also induced by androgens in prostate cancer cells [26], suggesting that EAF2 and ELL2 expression are induced coordinately by androgens in the prostate. Polyubiquitination of both EAF2 and ELL2 proteins could significantly enhance the dynamic range of EAF2/ELL2 expression levels in AR-positive prostate cells.

SIAH2 appears to be more potent than SIAH1 as an E3 ligase for EAF2 (Figure 5B). One limitation in our experiment was that the expression level of HA-SIAH1 in transfected HEK 293 cells was much lower than the transfected SIAH2 protein levels. The failure for HA-SIAH1 to co-immunoprecipitate EAF2 or to enhance the polyubiquitination of EAF2 may be due to the low level expression of HA-SIAH1 in the transfected cells. We cannot rule out the possibility for SIAH1 to act as an E3 ligase for EAF2. Although SIAH1 but not SIAH2 was reported as an E3 ligase for ELL2 by Liu, et al. the authors pointed out that the inability for SIAH2 to act as the E3 ligase of ELL2 was not a result of defective interactions between ELL2 and SIAH2 [15]. They proposed that different E2 ubiquitin-conjugating enzymes or cofactors are used by SIAH1 and SIAH2 in the catalysis of polyubiquitination and the lack of E2s/cofactors specific for SIAH2 may be responsible for the inability for SIAH2 to catalyze EAF2 polyubiquitination in our experiments. Polyubiquitination of EAF2 by SIAH2 but not SIAH1 may be due to the expression of E2s/cofactors required for SIAH2 but not for SIAH1 in transfected cells. It is possible that both SIAH1 and SIAH2 can catalyze polyubiquitination of both EAF2 and ELL2 in some types of cells.

The inhibition of EAF2 ubiquitination by ELL1 but not by ELL2 (Figure 6C and 6D) suggests that ELL1 and ELL2 are not functionally identical. ELL1 binding to EAF2 could significantly enhance EAF2 stability and expression level, which was consistent with ELL1 inhibition of EAF2 polyubiquitination (Figure 6A). In contrast, ELL2 binding to EAF2 had no significant effect on EAF2 polyubiquitination and did not seem to increase EAF2 stability in the presence of CHX (Figure 6B). Reciprocally, EAF2 also did not seem to increase the stability of ELL2. Thus, the EAF2/ELL2 complex is likely to be less stable than the EAF2/ELL1 complex. The unstable EAF2/ELL2 complex would allow quick down-regulation of EAF2/ELL2 proteins and their downstream signaling pathways in prostatic cells following androgen deprivation. This would facilitate prostatic cells to effectively respond to androgen deprivation.

SIAH2 was reported to increase in castration resistant prostate cancer and stimulate castration resistant activation of AR [27]. The elevated expression of SIAH2 may not only increase the transcriptional activity of AR, but also potentially reduce EAF2 protein level and its potential tumor suppressive activity, further promoting malignant growth of prostate cancer. Thus, inhibiting SIAH2 may also enhance EAF2 tumor suppressive activity, in addition to inhibiting castration-resistant AR activation.

## MATERIALS AND METHODS

### Antibodies and plasmids

The mouse EAF2 and EAF1 monoclonal antibodies were prepared as described previously [28]. Antibodies used included ubiquitin (sc-8017), AR (sc-816), PSA (sc-7638), GAPDH (sc-25778) from Santa Cruz Biotechnology (Santa Cruz Biotechnology, Santa Cruz, CA, USA). Antibodies to SIAH1 (NBP1-68088) and SIAH2 (NBP1-19648) were from Novus (Novus Biologicals, Littleton, CO, USA). Antibodies to PARP (#9532), Bcl-2 (#2876), survivin (#2803), tubulin (#2146) and Bax (#2772) were from Cell Signaling (Cell Signaling Technology, Beverly, MA, USA). Antibodies for myc (MMS-150) and HA-tag (MMS-101P) were from Covance (Covance, Berkeley, CA, USA), flag from Sigma-Aldrich (F1804, St. Louis, MO, USA) and GFP from Torrey Pines Biolabs (TP401, Houston, TX, USA). Anti-GFP mAb-Agarose was from MBL (MBL, Nagoya, Japan). Anti-myc agarose affinity gel and Anti-HA agarose affinity gel were from Sigma-Aldrich. N-ethylmaleimide (NEM, 04259), MG132 (Z-leu-leu-leu-al, C2211) and Cycloheximide (CHX, C7698) were from Sigma-Aldrich.

pCMV-myc-EAF2 and pEGFP-EAF2 plasmids were cloned as described previously [7]. HA-tag ubiquitin plasmid was a kind gift of Dr. Chunbing Zou from University of Pittsburgh. HA-tag SIAH1 and HA-tag SIAH2 plasmids were kind gifts of Dr. Qiang Zhou from University of California, Berkeley. The 17 single point mutations of EAF2 were introduced using a QuickChange II Site-Directed Mutagenesis Kit (Agilent Technologies) according to the manufacturer's instructions. EAF2 mutants with double or multiple point mutations were generated based on the single point mutant plasmids. All constructs were verified by sequencing (Genewiz Inc., Beijing, China).

### Cell culture

Human prostate cancer cells LNCaP and C4-2 were purchased from ATCC and provided by Dr. Leland W.K. Chung, respectively. These cells were maintained in RPMI 1640 with 10% fetal bovine serum (FBS), 1% Glutamine, 1% penicillin/streptomycin (Invitrogen, Carlsbad, CA) at 37°C in a humidified atmosphere containing 5% CO_2_. HEK 293 cells were obtained from ATCC and were maintained in DMEM supplemented with 10% fetal bovine serum (FBS). MG132 was added at 10–20 μM, 3 h before cell lysis. Transient transfections were carried out using Polyjet DNA Reagent (Signa Gen Laboratories, Rockville, MD, USA). Appropriate empty vectors were included as controls in all the transfections.

### Western blot

All Western analyses used whole-cell lysates unless indicated otherwise. Protein lysates were separated by sodium dodecyl sulfate polyacrylamide gel electrophoresis (SDS-PAGE), and transferred to a nitrocellulose membrane. Blots were blocked in Tris-buffered saline with 0.05% Tween and 5% nonfat milk followed by incubations with the indicated primary and secondary antibodies (Santa Cruz Biotechnology) in this buffer. Signals were visualized by enhanced chemiluminescence of ECL Western Blotting Detection Reagents (GE Healthcare Life Sciences, Piscataway, NJ, USA) and were exposed to X-ray film (Fuji film, Valhalla, NY, USA). Membranes were stripped between antibody probes using a stripping solution (β-Mercaptoethanol, 10% SDS, 0.375 M Tris pH 6.8).

### Fluorescent microscopy

C4-2 cells grown on coverslips were transfected with different combinations of mammalian expression vectors harboring GFP-EAF2, RFP-ELL1 or empty vectors. Forty-eight hours after the transfection, cells were fixed with 4% (w/v) paraformaldehyde at room temperature and blocked with 2% (w/v) bovine serum albumin in phosphate-buffered saline. The cells were then stained with DAPI (Life technologies, Grand Island, NY, USA) for 2 minutes. Images were acquired using a confocal microscope (Olympus, Fluoview-FV1000, Olympus America Co., Center Valley, PA, USA) using GFP, RFP or DAPI filter to detect co-localization of EAF2 and ELL1 in transfected C4-2 cells.

### Immunoprecipitation

HEK 293 cells were transiently transfected with 1–2 μg of plasmid DNA using Polyjet transfection reagent (SignaGen). Cells were separated into two aliquots, with one used in Western blot analysis to confirm expression of the transfected genes. The remaining cells were centrifuged and the cell pellets were washed twice in PBS, then resuspended in 600 μl to 1 mL NP-40 cell lysis buffer (20 mM Tris-HCl pH 7.5, 150 mM NaCl, 1.5 mM MgCl2, 0.5% NP-40, 15% Glycerol, 2 mM EDTA) containing a cocktail of protease inhibitors, incubated on ice for 30 minutes. To precipitate the complexes, supernatants were precleared with A/G agarose beads (Santa Cruz) for 1 hour at. Supernatants were incubated for 4 hours with 15–30 μL anti-myc beads or anti-GFP beads, rotated at 4°Cfor 4 hours, centrifuged for 3 minutes at 5000 g at 4°C. The pellets were then washed with a buffer containing 20 mM Tris-HCl pH 7.5, 250 mM NaCl, 1.5 mM MgCl2, 0.5% NP-40, 15% Glycerol, and 2 mM EDTA, boiled in 2× loading buffer for 5 minutes, fractionated by SDS–polyacrylamide gel electrophoresis (PAGE), and transferred to nitrocellulose membranes and analyzed by western blotting.

### EAF2 ubiquitination assay

HEK 293 cells or C4-2 cells cultured in 10 cm plates were co-transfected with 2 μg GFP-EAF2/myc-EAF2 plasmids and 4 μg HA-ubiquitin expression plasmids for 40 hours and then incubated with 10~15 mM MG132 for an additional 12–16 hours before harvesting. Cells were separated into two aliquots with one used in Western blot analysis to confirm expression of the transfected proteins. The remaining cells were used for purification of GFP-tagged/myc-tagged proteins by GFP-tagged/myc-tagged beads. The cell pellet was lysed in RIPA buffer with 5 mM N-ethylmaleimide and protein inhibitors on ice for 30 minutes. Supernatants were precleared with 30 μL of protein A/G agarose beads as indicated. The lysate was incubated with 20 μL anti-GFP/30 μL anti-myc beads and rotated at 4°C for 4 hours. The beads were washed with washing buffer containing 250 mM NaCl twice for 10 minutes and washing buffer containing 1 M NaCl (10 mM Tris-HCl pH8.0, 1 M NaCl, 1 mM EDTA, 1% NP-40) once for 10 minutes. Finally, the beads containing ubiquitin-conjugated proteins were boiled in 2× SDS loading buffer for 5 minutes and fractionated by 4–15% Ready Tris-HCl Gradient Gels (BioRad Laboratories, Hercules, CA). Samples were analyzed by western blotting.
