# Correction: Regulation of tumor suppressor EAF2 polyubiquitination by ELL1 and SIAH2 in prostate cancer cells

**DOI:** 10.18632/oncotarget.27354

**Published:** 2019-12-24

**Authors:** Xinpei Yu, Junkui Ai, Liquan Cai, Yifeng Jing, Dan Wang, Jun Dong, Laura E. Pascal, Jian Zhang, Rongcheng Luo, Zhou Wang

**Affiliations:** ^1^ Department of Urology, University of Pittsburgh School of Medicine, Pittsburgh, USA; ^2^ Department of Pathology, University of Pittsburgh School of Medicine, Pittsburgh, USA; ^3^ Department of Pharmacology and Chemical Biology, University of Pittsburgh School of Medicine, Pittsburgh, USA; ^4^ University of Pittsburgh Cancer Institute, University of Pittsburgh School of Medicine, Pittsburgh, USA; ^5^ Department of Geriatrics, Guangzhou General Hospital of Guangzhou Military Command, Guangzhou, China; ^6^ Department of Urology, Shanghai General Hospital, Shanghai Jiao Tong University School of Medicine, Shanghai, China; ^7^ Center for Translational Medicine, Guangxi Medical University, Nanning, Guangxi, China; ^8^ Cancer Center, Traditional Chinese Medicine-Integrated Hospital, Southern Medical University, Guangzhou, China; ^9^ Guangdong Provincial Key Laboratory of Geriatric Infection and Organ Function Support and Guangzhou Key Laboratory of Geriatric Infection and Organ Function Support, Guangzhou, China

**This article has been corrected:** Due to errors during image assembly, the RFP-ELL1 merged image in Figure 4B is incorrect. The proper Figure 4 is shown below. The authors declare that these corrections do not change the results or conclusions of this paper.

Original article: Oncotarget. 2016; 7:29245–29254. 29245-29254. https://doi.org/10.18632/oncotarget.8588

**Figure 4:**
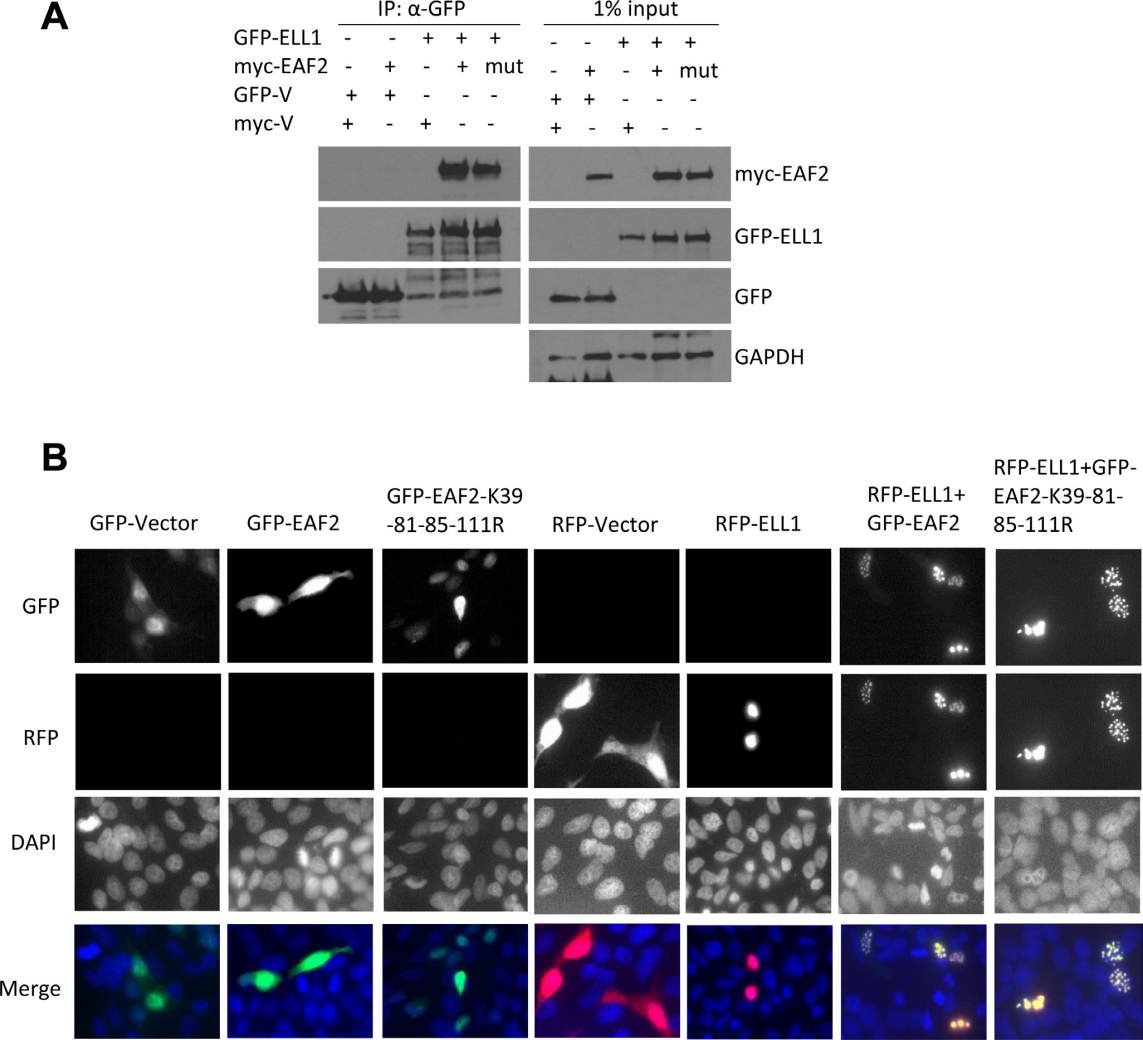
Mutant EAF2^K39-81-85-111R^ binding and co-localization with ELL1.

(**A**) HEK 293 cells were transfected with myc-EAF2, myc-EAF2^K39-81-85-111R^, or empty myc expression vector together with GFP-ELL1 or empty GFP expression vector for 36 h. The cell lysates were prepared for co-immunoprecipitation using anti-GFP antibody. The precipitates and whole cell lysates (1% input) were analyzed by immunoblotting using anti-myc and anti-GFP antibodies. GAPDH in the whole cell lysates was probed as loading control. (**B**) C4-2 cells were transfected with GFP, GFP-EAF2, GFP-EAF2^K39-81-85-111R^, RFP, and RFP-ELL1 expression vector alone or in the indicated combinations for 48 h. Subcellular localization was imaged with confocal microscopy. Image enlargement: 100×. Data shown are representative of three independent experiments.

